# Temperature-Induced Phase Transitions of Vivianite: In Situ Analysis of a Redox-Driven Crystallization

**DOI:** 10.1021/acs.inorgchem.5c02399

**Published:** 2025-08-28

**Authors:** Alice Paskin, Thaïs Couasnon, Roberts Blukis, Jeffrey Paulo H. Perez, Stefan Reinsch, Vladimir Roddatis, Marcin Syczewski, Liane G. Benning

**Affiliations:** † 28337 GFZ Helmholtz Centre for Geosciences, Telegrafenberg, 14473 Potsdam, Germany; ‡ Department of Earth Sciences, Freie Universität Berlin, Malteserstr. 74-100, 12249 Berlin, Germany; § Leibniz-Institut für Kristallzüchtung (IKZ), Max-Born Str. 2, 12489 Berlin, Germany; ∥ Federal Institute for Materials Research and Testing (BAM), Richard-Willstätter-Straße 11, 12489 Berlin, Germany

## Abstract

We document a solid-state, temperature-dependent (25–700 °C), multistage redox transformation of crystalline ferrous iron phosphate, vivianite (Fe_3_(PO_4_)_2_·8H_2_O). Under anoxic conditions, vivianite breaks down at *T* > 250 °C into an anhydrous, amorphous intermediate Fe_3_(PO_4_)_2_ phase, yet the bulk shape and morphology of the starting vivianite crystals were retained. This amorphous intermediate phase remained stable until *T* > 500 °C, after which a redox-dependent crystallization into two different minerals was observed. Under anoxic conditions, the amorphous ferrous intermediate (Fe_3_(PO_4_)_2_) transformed into the crystalline ferrous phosphate (graftonite, (Fe^2+^)_3_(PO_4_)_2_), while under oxic conditions it crystallized into a ferric phosphate (rodolicoite, Fe^3+^PO_4_). Graftonite formation occurs via an exothermic molar enthalpy (Δ*H*_cryst_) of −16.7 ± 0.2 kJ mol^–1^. Rietveld refinements of the two crystalline endmembers (vivianite and graftonite) revealed a unit cell volume decrease of ∼3.1% during the transformation, which was observed by in situ electron microscopic observations as an overall shrinking of the initial vivianite crystals. Despite volume loss and bubble-like features, the original vivianite shape was preserved, indicating a solid-state pseudomorphic transformation. Ex situ XRD and TEM-EELS analyses confirmed the ferrous-to-ferric oxidation, forming rodolicoite, through changes in the Fe geometry and oxidation state.

## Graphical abstract

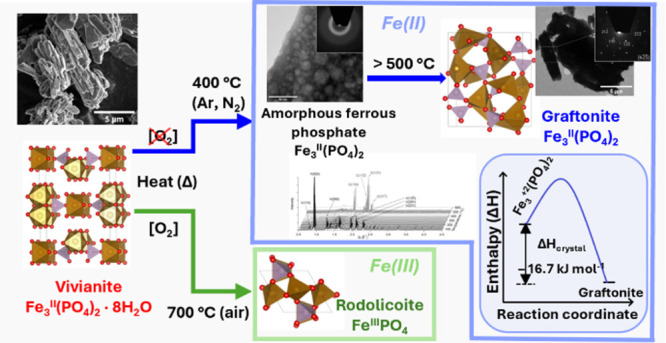

## 1 Introduction

Under anoxic aqueous conditions, at high phosphate and Fe^2+^ activities, the hydrated phase vivianite (Fe_3_(PO_4_)_2_·8H_2_O) is the most stable orthophosphate mineral.^1^ Vivianite occurs in nature at low temperatures in ferruginous lake and river sediments and soils.^1–3^ Meanwhile, synthetic vivianite has been of interest in energy technologies and is an economical precursor material for the synthesis of LiFePO_4_, a Li-ion battery cathode material.^4^ In such industrial processes, the morphology of the initial vivianite precursor can have a significant impact on the morphology and electrochemical activity of the synthetic LiFePO_4_ material,^4^ documenting the possible role of the morphology of synthetic vivianite in enabling the better design of optimum electrochemical cathode materials.

At ambient conditions, when exposed to air, vivianite will transform to meta-vivianite (Fe^2+^ Fe^3+^_2_ (PO_4_)_2_ (OH)_2_·6 H_2_O), due to structural Fe^2+^ oxidation.^5^ The oxidation of Fe^2+^ to Fe^3+^ in the vivianite structure has been proposed to occur via two potential pathways. First, by diffusion of oxygen through the parallel layers (*ac*) in its crystal structure, followed by the decomposition of crystal lattice water into hydroxyl ions coordinated to the Fe^3+^ sites.^6^ The second proposed pathway is via water auto–oxidation through decomposition of crystal water upon heating vivianite in vacuo under air.^6^ Although at room temperatures and anoxic conditions, vivianite can be stable for extended time periods, it can break down and or even oxidize rapidly during heating, and this ultimately influences the composition of the annealed products. On the other hand, at high temperatures, a naturally occurring crystalline and anhydrous ferrous phosphate encountered in granitic pegmatites is graftonite, a phase belonging to a class of minerals having a chemical composition of (M)_3_(PO_4_)_2_ (where M = Mn^2+^, Ca^2+^, and Fe^2+^), which can exist as a solid-solution series. Similarly, rodolicoite is a naturally occurring anhydrous crystalline ferric phosphate (Fe^3+^ PO_4_) mineral.^7^

Vivianite and graftonite are both ferrous phosphates that can co-occur in altered granitic pegmatites that have been subjected to hydrothermal alteration,^8^ yet the alteration parameters (e.g., temperature, pressure, and redox conditions) enabling the co-occurrence of vivianite and graftonite or what controls the intertransformation between vivianite, graftonite, and potentially rodolicoite are unknown. Experimentally, Nord et al. (1982) reported that heating a stoichiometric amount of amorphous ferrous phosphate (AFEP) for 1 month in an evacuated and sealed silica tube at 800 ± 10 °C led to the formation of synthetic graftonite.^9^ On the other hand, Huang et al. (2023) showed that heating synthetic amorphous ferric phosphate (Fe^3+^ PO_4_) in air at temperatures >620 °C also results in a crystalline ferric phosphate, rodolicoite.^10^ Both of these studies report heat-induced (stoichiometric) amorphous to crystalline transformations of an amorphous iron phosphate precursor.

From the crystallographic point of view, it is well-known that vivianite crystallizes in a space group *C2/m* with two distinct 6-fold coordination Fe sites.^11^ On the other hand, graftonite crystallizes in the *P21/c* space group and has a mixture of 5- and 6-fold coordination of Fe,^12^ whereas rodolocoite crystallizes in the *P3*_1_21 space group and has Fe^3+^ in tetrahedral 4-fold coordination.^10^ It is, therefore, clear that these crystalline phases bear very little similarity based on their local structure and symmetry, yet no evidence on the heating-induced conversion of vivianite to either of the two crystalline and high-temperature iron phosphate phases is available. These structural changes are accompanied by a preservation of the reduced ferrous iron in graftonite or a Fe oxidation state change in the case of rodolicoite. Yet none of these transformation reactions nor the mechanism of these intertransformations are known. To address this, we carried out experiments in which we followed the oxic and anoxic conversion of vivianite upon heating into various iron phosphate phases and characterized the reaction mechanisms based on data from a combination of in situ and ex situ experimental and analytical methods. Our experimental results uncovered a novel phase transformation mechanism of iron (ferrous/ferric) phosphates, shedding new light on the factors that govern their stability and compositional evolution.

## 2 Experimental Section

### 2.1 Vivianite Synthesis

Vivianite was synthesized inside an anaerobic chamber (Coy Laboratory Products, Inc.; 97% N_2_ and 3% H_2_), following our previously reported synthesis protocol.^13^ In brief, ultrapure water (∼18.2 MΩ·cm^–1^) was degassed by purging with CO_2_-free Ar gas and heating (at ∼80 °C, 6 h). This was used to prepare a Mohr’s salt solution ((NH_4_)_2_SO_4_·Fe­(SO_4_)·6H_2_O); 0.1 mol L^–1^, 50 mL, 99.95% purity, Alfa Aesar GmbH), reacted for 1 h under with a mixture of dibasic sodium phosphate (Na_2_HPO_4_; 0.0505 mol L^–1^, 25 mL, Sigma-Aldrich, 99.98%) and monobasic potassium phosphate (KH_2_PO_4_; 0.0494 mol L^–1^, 25 mL, Sigma-Aldrich). The experiments were performed in a 250 mL perfluoroalkoxy alkane (PFA) reactor. Vivianite precipitated immediately and was aged (1 week). The precipitates were vacuum filtered (0.2-μm filter, Nucleopore, Whatman) and washed with O_2_-free ultrapure water (30 mL). The resulting vivianite was a pale blue solid that was dried via overnight evaporation inside the anaerobic chamber and stored in airtight crimp seal vials. The as-synthesized vivianite was used as a starting material for various in situ and ex situ transformation experiments that were carried out both under anoxic and oxic conditions under heating. The experimental and characterization methods are detailed below.

### 2.2 Thermogravimetry and Differential Scanning Calorimetry (TGA-DSC)

At the start of our study, TGA-DSC test runs with vivianite revealed a high-temperature phase transformation that was highly air sensitive. Our initial data sets evidenced that the reaction pathway and final products varied significantly depending on the degree of anaerobicity in the system, and thus we were very careful to prevent any possibility of oxidation (refer to Section 3.1 for a discussion on this aspect). It is noteworthy that in these experiments, the anoxic vivianite samples were stored under nitrogen gas, transferred anaerobically and analyzed under argon gas in corundum crucibles to avoid oxidation. Fifteen mg vivianite crystals were weighed and placed in corundum crucibles within an anaerobic chamber and swiftly moved (<1 min) to the TGA-DSC instrument for analysis, minimizing air exposure. The TGA-DSC curves were simultaneously recorded on a thermobalance Themys One+ instrument (Setaram, Caluire, France), at a heating rate of 10 K min^–1^, 25–700 °C. Temperature and energy calibrations were performed by measuring the melting temperatures and enthalpy of fusion of pure metal standards in corundum crucibles. The metals In, Al, Ag, Au, and Ni were used for temperature calibration (error ± 0.6 °C), and In, Sn, Al, Ag, and Au were used for energy calibrations. The data was evaluated with the Themis One+ software (Setaram, Caluire, France). At the end of each measurement, the resulting solids were retrieved for further characterization.

### 2.3 Powder X-ray Diffraction (XRD) and In Situ High Temperature XRD (HT-XRD)

The phase identification and structural analyses were performed via powder XRD measurements. Samples were loaded into quartz glass capillaries (Hilgenberg, 80 × 0.5 mm) and sealed with gum sealant (Cristaseal, Hawksley & Sons) inside the anaerobic chamber. XRD patterns were recorded on a STOE STADI P diffractometer (STOE & Cie GmbH, Germany) with a curved Ge(111) monochromator and DECTRIS MYTHEN2 R detectors in Debye–Scherrer geometry (40 kV, 40 mA) using Ag Kα radiation (λ = 0.55941 Å), a 0.015° step size, and 2500 s per step. An empty capillary measured under identical conditions served as background. Calculated XRD patterns were generated using VESTA,^14^ and qualitative phase identification was performed with QUALX2 (v 2.1).^15^

In situ HT-XRD measurements were performed by using a STOE furnace accessory HT1. The furnace accessory was temperature-calibrated (error ± 0.58 °C) by using KNO_3_ (Alfa Aesar, >99%) and KCl (Sigma-Aldrich, >99%) standards at a heating rate of 10 °C min^–1^. The calibration curves are shown in Supporting Information Figure S1. Vivianite heating experiments and measurements were performed across four temperature ranges (10 °C min^–1^). This way, we optimized the acquisition time and prevented vivianite oxidation within the capillary via rapid initial dehydration: these were (I) 25–400 °C (range 1), (II) 400–550 °C (range 2), (III) 450–550 °C (range 3), and (IV) 550–600 °C (range 4). XRD patterns were recorded in Debye–Scherrer mode (0 to 73°, 0.015° step size (180 s per step)) as a function of temperature. The samples were prepared and sealed using quick-sealing glue (Bolton Adhesives) into quartz glass capillaries inside the anaerobic chamber. The XRD patterns over the different temperature ranges were compiled to construct in situ plots across the temperature range between 25 and 600 °C. For visual inspection and observation, the relative intensities of the patterns were scaled to improve the *signal-to-noise* ratio. The room temperature powder XRD and the in situ XRD patterns collected at different temperatures were refined via Rietveld analyses on GSAS-II software^16^ starting with initial models for vivianite^17^ and Fe-graftonite^18^ with phase fraction analyses at fixed histogram scale factors (=1). Background estimation was done via Chebyschev polynomial fitting, and samples were modeled as pure phases. The unit cell parameters, microstrain, grain size, thermal displacement, and atomic coordinates were the refined parameters. The NIST 660c LaB_6_ standard was used to calibrate the instrument line profile. The normalized factors (α) were then plotted as a function of the temperature.

### 2.4 Conventional and In Situ Heating Scanning Electron Microscopy (SEM)

The morphologies and qualitative elemental composition of the synthesized initial vivianite and the solid products following the temperature-dependent transformation experiments from the TGA-DSC and XRD runs were imaged and further analyzed using a field emission gun scanning electron microscope (FEG-SEM, FEI Quanta 3D, run at a voltage of 20 kV and 83.3 pA current) coupled to an energy dispersive X-ray spectrometer (EDX, Octane Elect EDAX detector). Prior to insertion into the microscope, the solids were dispersed onto sticky carbon pads that had been glued to the SEM aluminum stubs and carbon coated (∼20 nm layer) with a Leica EM ACE600 high-vacuum sputter coater. The SEM-EDX relative peak areas were analyzed with EDAX-TEAM software (AMETEK Inc.).

We also performed in situ SEM heating experiments using an in situ transmission Kikuchi diffraction (TKD) stage (DENS solutions) dedicated specimen holder by depositing vivianite powders on a heating chip and imaging transformations at an acceleration voltage of 20 kV, 1 mbar of N_2_, from 23 to 700 °C and using a heating rate of 10 °C min^–1^ to match the TGA-DSC and XRD measurement conditions.

### 2.5 Heat-Induced Transformation of Vivianite in Air

To study the oxidation behavior of vivianite under oxic conditions, ∼1 g of the vivianite powder was placed into a ceramic crucible and heated in air inside a Thermolyne benchtop muffle oven (Thermo ScientificTM) to 700 °C for 2 h. At the end, a reddish-yellow powder was obtained that was characterized by XRD.

### 2.6 Infrared Spectroscopy (IR)

The samples were analyzed using IR spectroscopy to gain insights into the bonding environment and functional group bonding environments (water and phosphate). Fourier transform IR patterns of the dried solids were measured at room temperature (25 °C) in attenuated total reflection (ATR) mode on a Nicolet iD5 spectrometer (Thermo Fischer Scientific, USA) with a single bounce diamond iD7 ATR accessory. For each FTIR pattern, 16 scans were averaged, and data was recorded at a resolution of 4 cm^–1^.

### 2.7 Transmission Electron Microscopy (TEM)–Electron Energy Loss Spectroscopy (EELS)

High-resolution imaging, selected area electron diffraction (SAED), and EDS analyses of the solids were performed by TEM (TECNAI F20 XTWIN) operated at 200 kV, with a field emission gun electron source and a Gatan Imaging Filter (GIF) Tridiem EDAX X-ray analyzer. The TEM-EELS measurements were performed at ambient temperature (25 °C) using a Themis Z (3.1) scanning transmission electron microscope equipped with a Gatan Continuum 1065ER spectrometer on powders of crystalline ferric phosphate, AFEP, and graftonite, which were drop casted onto a lacey carbon film supported by a Cu grid.

### 2.8 X-ray Absorption Spectroscopy (XAS)

The Fe K-edge XAS data were collected on the P65 undulator beamline of a DESY German Electron Synchrotron (HASYLAB, DESY PETRA III, Hamburg, Germany). The spectra were recorded at room temperature in transmission mode to a reciprocal space value of ∼14.5 Å^–1^. The details of sample preparation, protocols to prevent oxidation during sample transport, and XAS beamline details are described in the Supporting Information Section S1. Spectra were aligned, averaged and background subtracted using the Athena software.^19^ The normalized X-ray absorption near edge structure (XANES) was exported to OriginPro software (Origin Laboratories©, 2021). Shell-by-shell fits were performed on the *k* ^3^-weighted extended X-ray absorption fine structure (EXAFS) using the SIXpack software^20^ based on an algorithm derived from IFFEFIT.^21^ Detailed information on EXAFS analysis can be found in Supporting Information Section S1.

## 3 Results and Discussion

### 3.1 High-Temperature-Induced Phase Transformations of Vivianite upon Anoxic Heat Treatment

The synthesized vivianite was a pale blue solid, characterized via powder XRD and IR analysis. Its XRD pattern showed crystalline Bragg reflections, which could be indexed (Figure S2 Supporting Information) based on the existing crystal structure of vivianite.^17^ Considering that vivianite can oxidize relatively fast, we have taken all measures to prevent its oxidation during our analyses. For example, XRD analyses were carried out with samples sealed inside an anaerobic chamber in capillaries, while for TGA/DSC analyses, samples were prepared and transferred to crucibles inside an anaerobic chamber, stored in airtight vials, and exposed to air for less than 1 min during transfer to the instrument. Once loaded, the TGA-DSC chamber was evacuated and purged with Ar gas to maintain anoxic conditions. Our previous work showed that such brief air exposure during transfer does not cause detectable oxidation of vivianite.^13^

The anoxic TGA-DSC analysis of this sample from 25 to 700 °C revealed stepwise loss of the volatile component (H_2_O) from its structure (Figure 1). The patterns of the TGA-DSC analysis of the vivianite (Fe_3_(PO_4_)_2_·8H_2_O) heating showed a major mass loss of ∼18% between 100 and 200 °C, representing a loss of 63% of the stochiometric H_2_O of vivianite (∼5 mol), highlighted as the gray regime in Figure 1. This mass loss equates to the dehydration reaction:

$${\mathrm{Fe}}_{3}({\mathrm{PO}}_{4}{)}_{2}\cdot 8H_{2}O_{\left(s\right)}\to {\mathrm{Fe}}_{3}({\mathrm{PO}}_{4}{)}_{2}\cdot 3H_{2}O_{\left(s\right)}+↑5H_{2}O_{\left(g\right)}$$ 1

**Figure 1:**
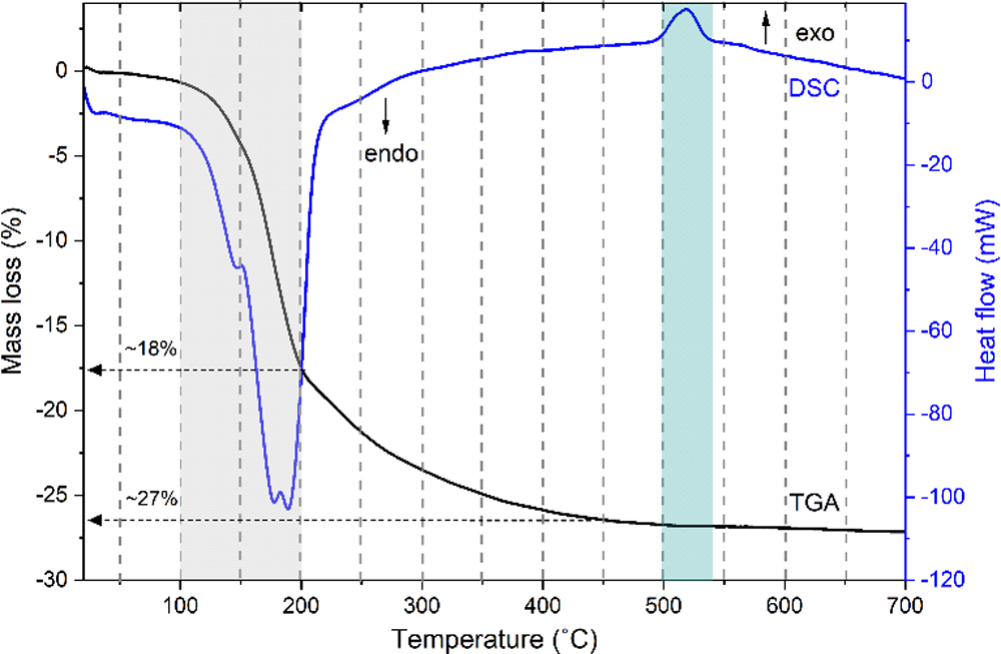
TGA (black) and DSC (blue) curves of vivianite for a heating rate of 10 °C/min from 25 to 700 °C. The gray area indicates the major endothermic mass loss step (∼18%) between 100 and 200 °C, and the blue area indicates the exothermic peak (∼27%) with an onset temperature of 505 °C, a peak maximum at 518 °C, and an offset temperature of 540 °C (with no observable mass loss).

This reaction was accompanied by a sharp endothermic DSC signal (Figure 1 blue curve), typical for a dehydration process.^22^

There is a lower rate of H_2_O mass loss (∼27%) continuing up to ∼450 °C, representing its complete dehydration (∼8 mol H_2_O) of vivianite, giving rise to a final material with the composition Fe_3_(PO_4_)_2_. With continued heating, there were no further observable changes in the TGA pattern from 450 to 700 °C (end of TGA-DSC run), showing that the Fe_3_(PO_4_)_2_ material remained stoichiometrically intact. These findings are (for the most part) consistent with the TGA-DSC analysis of natural vivianite by Frost et al.^23^ However, it is interesting to notice an exothermic peak in the DSC curve between ∼500 and 540 °C, unaccompanied by mass loss, suggesting a spontaneous structural modification. Similar exothermic effects in TGA-DSC spectra have been attributed to the transformation of anhydrous amorphous calcium phosphate (ACP) → HAP (hydroxyapatite) in the Ca-PO_4_ system.^24^

The presence of this exothermic peak, therefore, implies a solid–solid transformation^24^ and recrystallization of the dehydrated vivianite. Furthermore, the DSC data could be used to calculate the molar enthalpy of this transformation (Δ*H*_cryst_) as −16.8 ± 0.3 kJ mol^–1^ from the precalibrated DSC data. The calculated entropy change associated with this transformation is 21.5 J K^–1^ mol^–1^, reflecting the increased structural order as the material undergoes a transition from the disordered amorphous phase to the more highly ordered crystalline phase.

These transformations are also invariably linked to structural changes in the solid phases. To document this, we performed in situ powder XRD experiments between 25 and 600 °C (Figure 2). The vivianite powder was sealed in a capillary under an anoxic atmosphere, and the high-temperature XRD furnace was purged with nitrogen during the measurements to prevent oxidation through air diffusion. Partial oxidation of vivianite can produce a mixture of ferrous and ferric phosphate phases, complicating the analysis. Therefore, it is important to maintain anoxic conditions throughout. The patterns revealed the presence of only crystalline vivianite between 25 and 120 °C. Upon continued heating, a gradual decrease in the relative intensities of vivianite’s Bragg reflections was observed because of dehydration (Figure 2) as inferred from TGA-DSC data. At ∼200 °C, all of the Bragg reflections corresponding to crystalline vivianite, including the most visually prominent (010) reflection at 0.95 Å^–1^, representing the Fe–P sheets interlinked with hydrogen bonds between lattice water molecules, could no longer be discernible. This decrease matched the dehydration documented from our TGA-DSC data (Figure 1) all the way to ∼200 °C; while in the in situ XRD, the loss of all the Bragg reflections corresponding to crystalline vivianite indicates the collapse of the vivianite interlayers. Between ∼200 and 500 °C, the in situ XRD patterns contained no sharp Bragg peaks and showed broad scattering features, indicating the breakdown of the vivianite crystal structure (Figure 2). This result was cross confirmed by ex situ XRD analyses of solids harvested from a TGA/DSC experiment, where the sample was heated in a crucible under Ar gas (up to 400 °C) and analyzed by powder XRD (Figure 3), showing broad features representative of a poorly ordered or amorphous material.

**Figure 2:**
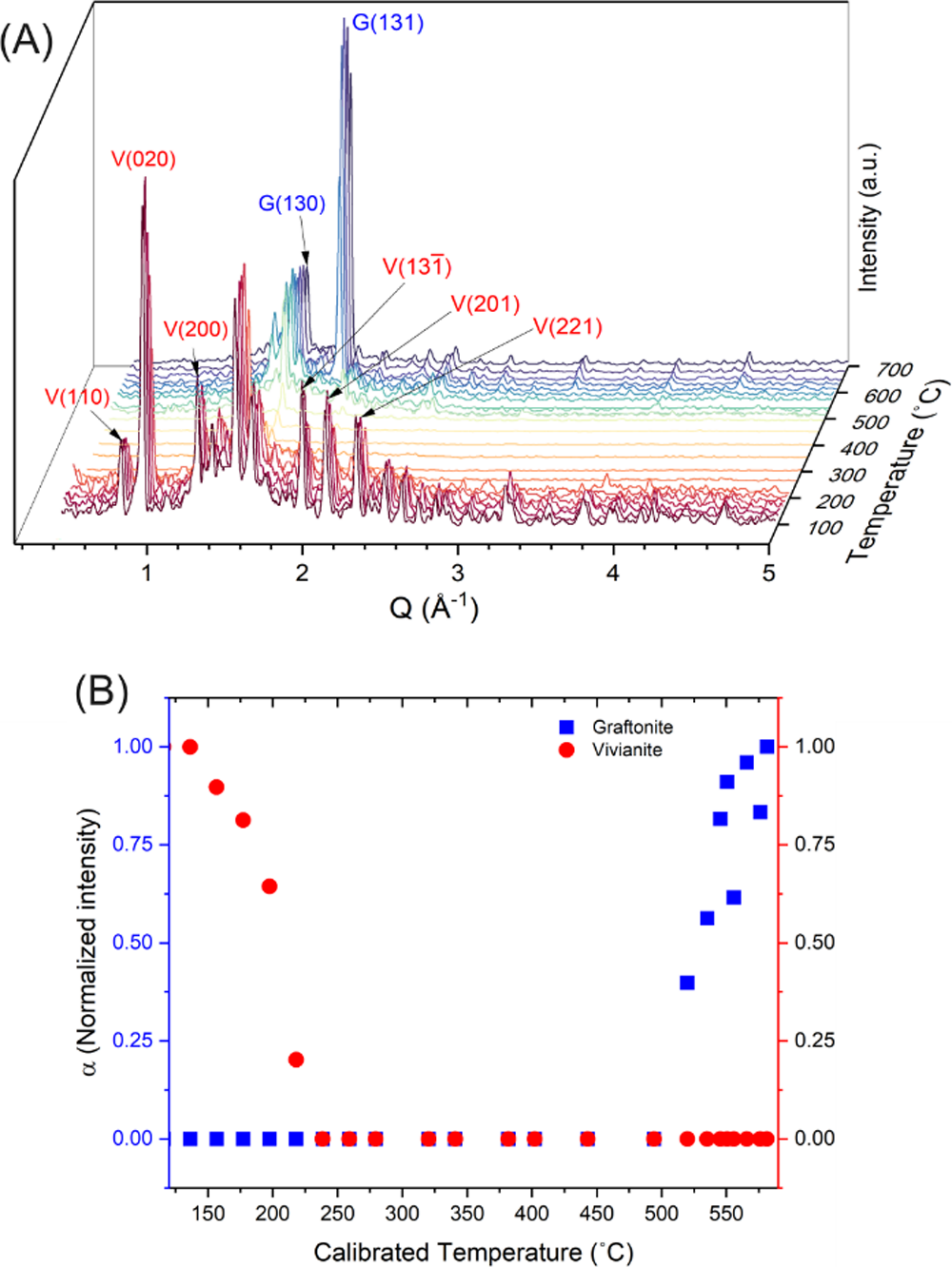
(A) In situ powder XRD diffractograms showing the anaerobic, dry breakdown of vivianite first to an amorphous phase <∼200 °C and its subsequent recrystallization into graftonite >∼500 °C. The respective Bragg reflections have been assigned to vivianite (V) and Fe-graftonite (G), respectively; (B) plots of α (normalized phase fractions (PF) from Rietveld refinements) as a function of (calibrated) furnace temperature. Color schemes: red—vivianite; blue—graftonite.

**Figure 3:**
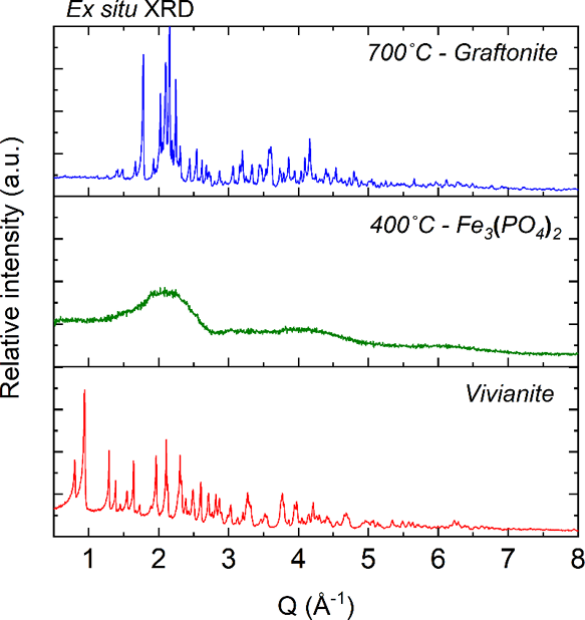
Ex situ XRD patterns of vivianite and powders collected from TGA-DSC crucibles (under Ar gas) at 400 (amorphous phase) and 700 °C (graftonite). The XRD measurements were performed at ambient temperature (25 °C).

With further heating, first evidence of a thermally induced recrystallization of the amorphous phase was documented in the XRD patterns at ∼500 °C. This was evidenced by the rapid increase in sharp Bragg reflections (Figure 2) that could be assigned to the Fe-graftonite structure reported by Henry et al.^18^ The most relatively intense (130) and (131) Bragg reflections of graftonite were initially observed at *Q* values of 1.83 and 2.20 Å^–1^ (Figure 2) just >500 °C, followed by their steady growth until the end of the experiment at 600 °C, where no further changes in the XRD patterns were observed. These results agreed with our TGA-DSC data (Figure 1), which indicated that a crystalline but anhydrous Fe_3_(PO_4_)_2_ phase formed at this temperature, as evidenced by the exothermic peak in the TGA-DSC curves.

Combining the TGA-DSC data (Figure 1) with the time-resolved in situ XRD data (Figure 2) allowed us to unequivocally document that under dry, anoxic conditions the thermally induced transformation reaction proceeded via the breakdown of the initial vivianite crystal structure into an amorphous ferrous phase, followed by its recrystallization to graftonite. This was further cross-confirmed by ex situ XRD patterns and Rietveld refinements (Figure 3) that allowed us to determine the unit cell parameters of the crystalline end-members (initial vivianite and end product Fe-graftonite), which could be very well fitted with the literature data for pure phase Fe-graftonite by Henry et al.^18^ and vivianite by Fejdi et al.,^17^ respectively.

#### 3.1.1 Rietveld Refinements for Quantification of Cell Parameter Changes during This Transformation

The Rietveld refinement results (Table 1 and Figure 4) revealed that although vivianite and graftonite crystallize as centrosymmetric monoclinic Bravais lattices, there are variations in their space groups. Structurally, vivianite is crystallized in *C2/m*, while graftonite is crystallized in the *P21/c* space group.

**Figure 4:**
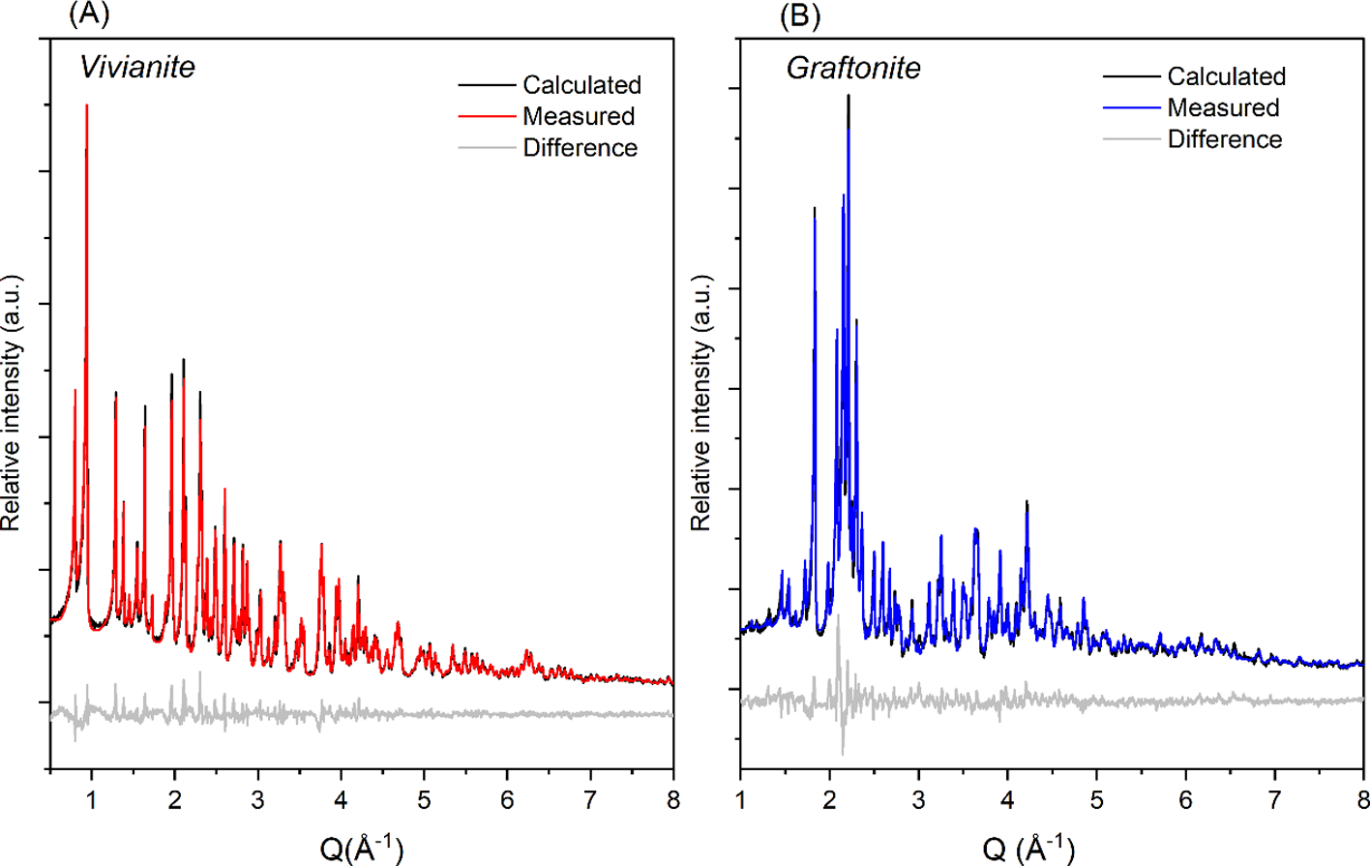
Rietveld refinement results for (A) synthetic vivianite—red trace and (B) graftonite—blue trace (anoxically heat-treated vivianite harvested from TGA-DSC run up to 700 °C) showing the measured (at ambient temperature), calculated, and difference powder XRD patterns.

**Table 1:** Details of Rietveld Refinements of Powder XRD Data (Ex Situ—25°C) for Synthetic Vivianite and Graftonite from This Work

phase	space group	a (Å)	b (Å)	c (Å)	β (°)	volume (Å^3^)	R_wp_ (%)
vivianite	*C 2/m*	10.09 (17)	13.43 (6)	4.70 (4)	104.30 (28)	618.94 (5)	5.45
graftonite	*P 21/c*	8.87 (18)	11.16 (10)	6.13 (10)	99.33 (6)	599.60 (12)	7.56

The unit cell volume decreases by 3.11 ± 0.011% upon heat-induced dehydration of vivianite, followed by amorphization to anhydrous Fe_3_(PO_4_)_2_ and its crystallization to the Fe-graftonite structure. This also implies a density change. Theoretical calculations from literature values of unit cell volumes from the structural models of vivianite^17^ and Fe-graftonite^18^ predict a volume decrease of 3.19%, which matches the volume decrease calculated from our data.

Additionally, there are also changes in coordination environments of the two crystalline end-phases, Fe^2+^ changes from distorted octahedrally coordinated in vivianite (coordination no. (CN) = 6), to a mixture of trigonal bipyramidal and distorted octahedral (CN = 5 and 6) in graftonite. Because the local structure of the intermediate anhydrous amorphous Fe_3_(PO_4_)_2_ could not be ascertained by XRD and Rietveld refinements, we explored the nature and chemical characteristics of this anhydrous amorphous transformation intermediate together with its crystalline end products ex situ by analyzing their local bonding environments (FTIR spectroscopy), chemical composition (SEM-EDS analyses), oxidation states, and Fe coordination (XANES/EXAFS) and morphology (TEM/SEM images).

#### 3.1.2 Structural and Morphological Changes upon HT-Treatment of Vivianite

The FTIR spectra provided structural insights into the difference in water and phosphate stretching and bending regions for the initial vivianite and all solids recovered after the TGA-DSC runs (Figure 5) and indicated that all three phases differ in structure and hydration. For the initial vivianite, the water hydroxyl stretching (ν_O–H_) region (∼3500–2500 cm^–1^) contained more than one vibrational peak, indicating the different water bonding environments in its crystal structure. The characteristic water bending mode (δ_H–O–H_) in vivianite appeared as a medium and broad peak at ∼1635 cm^–1^, while the water librational mode is a sharp and intense peak at ∼810 cm^–1^, correlating well with the literature value.^13,25^ The FTIR spectra of the amorphous intermediate phase and product graftonite obtained from the TGA-DSC runs at 400 and 700 °C confirmed that both of these phases were indeed anhydrous as evidenced by the absence of stretching and bending vibrations related to water and the very weak absorbance in the O–H stretching and bending region. The water librational mode at 810 cm^–1^ also disappears in the case of anhydrous Fe_3_(PO_4_)_2_ and Fe-graftonite, as expected from the loss of water from their local structure. The characteristic stretching vibrational modes for phosphate (ν_s_ and ν_as_) were present in the same region for all three phases (∼1050–800 cm^–1^). However, differences in the bonding environments and structural arrangements are notable through the changes observed in the splitting of these bands.

**Figure 5:**
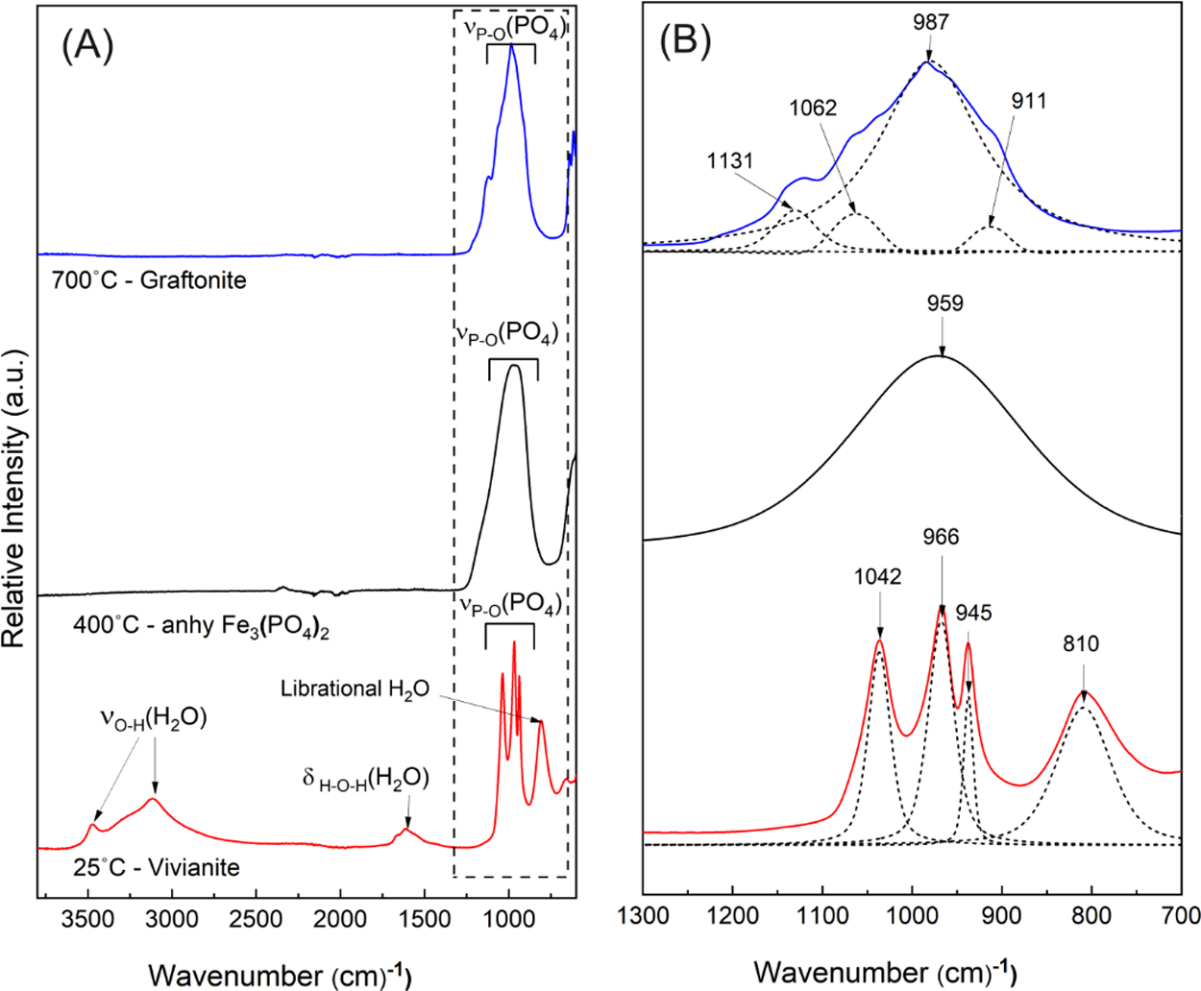
Ex situ FTIR spectra of vivianite—red trace, the anhydrous, amorphous intermediate Fe_3_(PO_4_)_2_ produced at 400 °C,—black trace, and the crystalline end product from 700 °C, graftonite–blue trace; the solid materials were harvested from anoxic TGA-DSC runs (under Ar gas); (B) Figure shows a zoom of the dotted area in (A) showing the P–O symmetric and asymmetric stretching vibrations (1200–900 cm^–1^) and H_2_O librational mode for vivianite (810 cm^–1^) deconvoluted into pseudo-Voigt components for the different phases.

As such, vivianite has three sharp phosphate group peaks which could be fitted with three pseudo-Voigt peaks centered at 1042, 966, and 945 cm^–1^ due to the nondegeneracy of vibrational energy levels of the PO_4_ groups in its crystal structure (Figure 5). Contrastingly, the anhydrous and amorphous Fe_3_(PO_4_)_2_ is characterized by a single, broad vibrational peak for the phosphate P–O stretching centered at 959 cm^–1^ that is merged into one band. We have shown previously,^13^ partly also by FTIR analyses, that when vivianite crystallizes from solution at ambient temperatures through a nonclassical pathway, it also forms via an intermediate, but partially hydrated AFEP (Fe_3_(PO_4_)_2_·∼4.7H_2_O). This vivianite precursor AFEP contains ∼4.7 molecules of water and is also characterized by a single broad P–O stretching peak centered at 976 cm^–1^.

Importantly, in the FTIR spectrum of partially hydrated AFEP, this P–O stretching peak is shifted by ∼17 cm^–1^ to lower wavenumbers, compared to the anhydrous AFEP phase formed during the dry breakdown of vivianite at 400 °C. This shift reveals differences in P–O bonding environments, potentially due to absences of H-bonding in the anhydrous intermediate compared to the partially hydrous AFEP.

On the other hand, the FTIR spectrum of graftonite showed a sharp peak which could be fitted with four Pseudo-Voigt components for the P–O (asymmetric and symmetric) stretching vibrations centered at 987 cm^–1^ and medium shoulders at 1131, 1062, and 911 cm^–1^, reflecting its crystalline nature. Overall, our ex situ FTIR data (Figure 5) confirm the expected differences in (vibrational) local structures and bonding environments in the three phases (vivianite, anhydrous amorphous Fe_3_(PO_4_)_2_, and Fe-graftonite) in our anoxic system.

To ascertain the nanoscale variation in the atomic structure in these phases, ex situ TEM analysis coupled to SAED was performed. The solids collected at 400 °C showed a porous, noncrystalline material based on broad scattering from its associated SAED pattern (Figure 6). The TEM micrographs showed the presence of nonuniform circular cavities or holes. The material was dehydrated and anhydrous prior to imaging and analysis (as confirmed by the TGA and IR data), and we avoided beam damage related to prolonged exposure to the electron beam. On the other hand, TEM analysis of the samples harvested from the TGA-DSC run under Ar gas at 700 °C showed aggregated crystals of Fe-graftonite ∼0.5 μm in size. The SAED pattern (Figure 6B, inset) revealed a monocrystalline domain of graftonite based on the indexed pattern from the crystallographic information on Fe-graftonite.^18^ The TEM data agree with the results from the XRD and FTIR data. As seen in Figure 6A, the pores/bubble-like features were distributed nonuniformly within the sample and varied in diameters, with the surface showing smaller pores compared to the internal bulk. At the graftonite-air interface or the crystal surface, the minimization of surface energy (via smoothing/reduction of pore size) during heating may be a contributing factor leading to variations in pore-size distribution on the sample surface versus bulk, suggesting that these features may be due to dehydration and not beam induced.^13,26^

**Figure 6:**
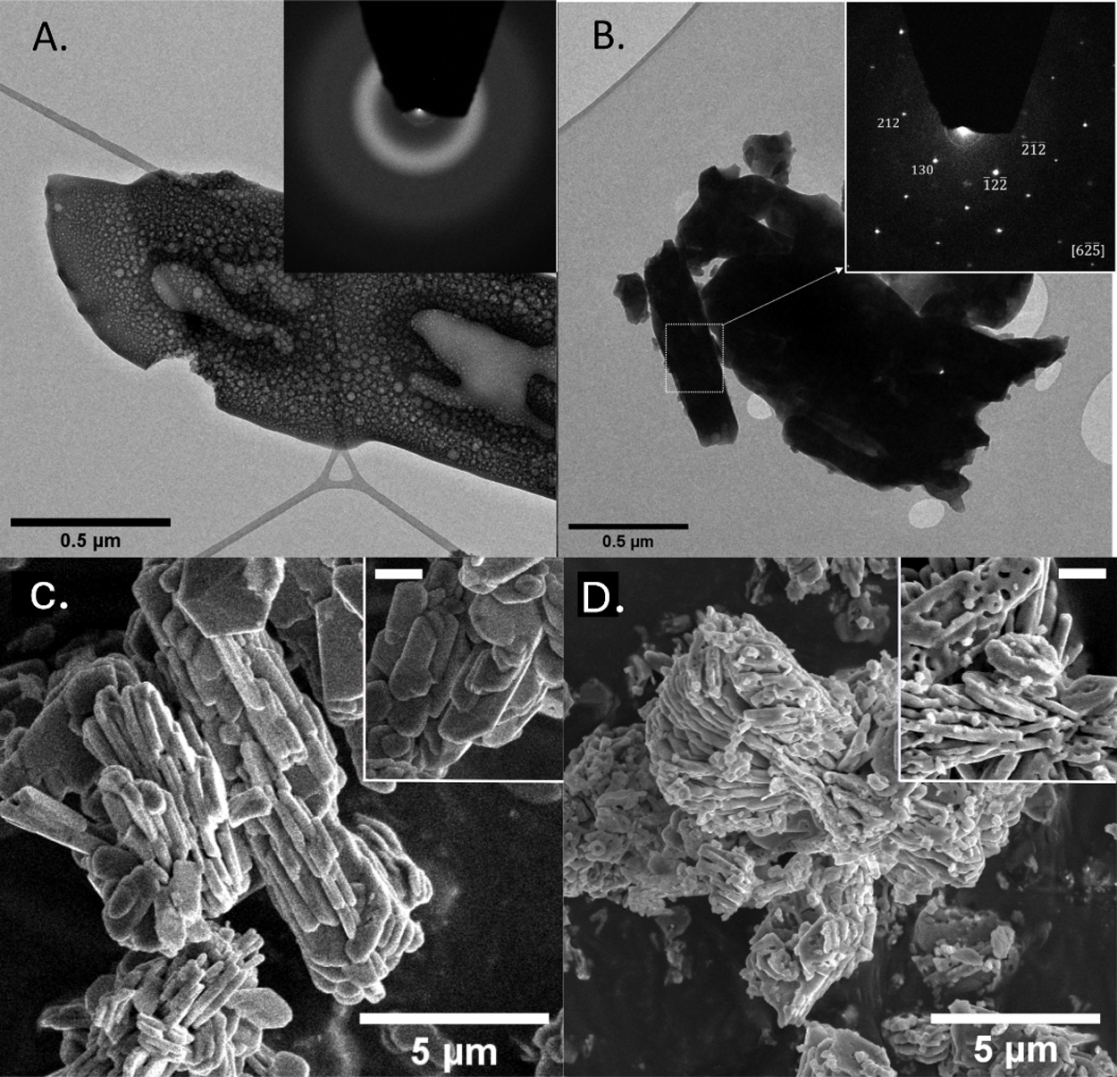
Ex situ TEM micrographs and associated indexed selected area electron diffraction (SAED) patterns for (A) Fe_3_(PO_4_)_2_ (400 °C) and (B) graftonite (700 °C); SEM micrographs of (C) vivianite showing smooth crystal surfaces and (D) graftonite showing a rough surface texture. (The white bar in the *inset* represents a scale bar of 100 nm).

The morphologies and surface habits of the two crystalline end phases, vivianite and graftonite, when imaged ex situ via SEM (Figure 6C,D), revealed tabular crystals within a size range of 2–5 μm. Vivianite occurred as aggregated crystal platelets, with the surface of each crystalline platelet being smooth. On the contrary, the surface morphology of the Fe-graftonite platelets was not smooth but rough and porous. The presence of these surface features may be directly linked to the pores/bubble-like features in the annealed products, as observed in TEM micrographs (Figure 6A,B,D—inset). During the in situ SEM experiment (Figure S4, Supporting Information), we observed most of the transformation effects detected by other ex situ methods (TEM, SEM, and XRD), namely: shrinking of initial vivianite crystal agglomerates (as evidenced by calculated volume decrease based on XRD Rietveld refinements) and formation of “bubble”-like contrast features (Figure 6).

These surface features on graftonite indicate that during the crystallization, the original vivianite morphological features have been transferred, and it is likely that the transformation proceeded via a solid-state mechanism. As seen in the in situ SEM image snapshots and the video (Figure S3, Supporting Information), the overall shapes of the initial vivianite crystals were preserved during heating and amorphization as well as during the subsequent recrystallization to graftonite. We also noted a rapid shrinking of the particle volumes during the recrystallization to graftonite (Video 1, Supporting Information).

Note that the temperatures where we observed fast changes during the *in situ* SEM image recording (Video 1 and Figure S3, Supporting Information) differed from those recorded during the in situ TGA-DSC and in situ XRD transformations (Figures 1 and 2). This is because the temperature in the SEM sample chamber was set at the contact between the crystals on the sample holder, and images are collected from the surface of the observed crystals (Figure S3, Supporting Information). Thus, the temperatures noted in the image snapshots and video shown in the Supporting Information file from the in situ SEM transformation experiments are lower, and only the relative differences between images should be considered.

Nevertheless, as expected from all other data sets, the EDX data of both the initial vivianite and final graftonite contained major peaks for Fe, P, and O (Figure S4, Supporting Information). The Fe and P peaks were of similar relative intensities. The calculated Fe:P peak area ratio of 1.45 for vivianite and 1.51 for Fe-graftonite are also within EDX error of the theoretical Fe:P ratio of ∼1.5 for both these phases.

To evaluate if any changes in oxidation states and coordination environment of iron occurred during the anoxic experiments, we performed Fe–K edge XANES analysis on vivianite, the anhydrous, amorphous intermediate, and crystalline graftonite. The position of the first-derivative maximum of the Fe K-edge XANES spectra of graftonite is at ∼7121 eV (Figure S5, Supporting Information), which matches the maxima of Fe^2+^ reference phases such as vivianite and that of hydrated amorphous intermediates such as AFEP^18^ (Figure S5, Supporting Information). Furthermore, the shell-by-shell fitting of the Fe K-edge EXAFS spectra allowed determination of the local Fe bonding environment (coordination number, CN) and bond distances (*R*) of the different phases (see Supporting Information Figure S6 and Table S3). The first neighbor contribution to the EXAFS spectra of the vivianite is best fitted with CN ∼ 6.7 and an Fe–O bond distance (*R*_Fe–O_) of 2.09 Å. The CN and *R* values of the anoxic amorphous intermediate - Fe_3_(PO_4_)_2_ (CN_Fe–O_ ≈ 4.2, *R*_Fe–O_ = 2.03 Å) are lower than those of the AFEP (CN_Fe–O_ ≈ 6.4, *R*_Fe–O_ = 2.13 Å),^13^ a partially hydrated AFEP phase that forms prior to the crystallization of vivianite in solution. Similarly, the first shell Fe–O of graftonite (700 °C) has the lowest CN value of ∼3.7 and the shortest *R*_Fe–O_ of 1.99 Å. The lower amplitude (and thus CN value) of the transformed samples could be caused by potential destructive interferences with Fe–O scattering waves due to the large distortions in the three distinct Fe–O polyhedra. The decreasing *R*_Fe–O_ upon anoxic heat treatment can be attributed to a greater mass percentage of phosphorus in the heat-treated samples, relative to the hydrated phases (vivianite and AFEP), and therefore higher Fe → oxygen → phosphorus π back bonding interactions.^13^

#### 3.1.3 Changes in the Mineralogy of This Transformation in the Presence of Oxygen

It is essential to note that all heating experiments discussed so far were performed in the absence of oxygen and under an inert atmosphere (i.e., TGA-DSC—Ar gas, XRD—N_2_ gas, SEM—N_2_ gas) to avoid any potential oxidation of Fe^2+^ to Fe^3+^ in the initial vivianite samples. After these anoxic transformations, our analyses of the resulting graftonite (a Fe^2+^ compound) exposed to air for up to 1 month indicated that the crystal structure (XRD pattern) and local bonding environment (IR spectrum) remained unchanged, agreeing to literature results.^27^ This suggests that once the anoxically heated vivianite transformed to graftonite (>500 °C), the transformed product was no longer susceptible to aerial oxidation.

Vivianite oxidation is influenced by the air exposure and temperature. Mössbauer studies indicate that under ambient conditions, oxidation continues until about 50% of structural Fe^2+^ is oxidized.^28^ Treating a vivianite suspension at 50–60 °C leads to meta-vivianite formation.^29^ DFT studies reveal that the phase transformation from vivianite to meta-vivianite is driven by better accommodation of H-vacancies and defects in meta-vivianite’s structure, whose formation becomes thermodynamically feasible once 66% of Fe^2+^ in vivianite is oxidized, highlighting the intricate relationship between oxidation levels and structural stability. A natural question arises: Does ferrous vivianite transform into a new phase when it is heated in air at high temperatures? Does the Fe speciation also change if the transformation produces a crystalline high-temperature phase? Thus, to determine the effect of oxygen on the transformation, we heated synthetic vivianite in a furnace up to 700 °C for 1 h under oxic conditions. This oxic heat-treatment surprisingly yielded a crystalline reddish-yellow powder, instead of the gray-colored graftonite. The XRD pattern of the yellow powder could be fitted to the theoretical pure crystalline ferric phosphate phase rodolicoite^7^ (Figure 7). Rodolicoite is a ferric iron phosphate (Fe^3+^PO_4_) phase with a structure completely different from that of vivianite or graftonite. Rodolicoite has a tetrahedral coordination of Fe atoms and crystallizes in the *P3*_1_21 trigonal space group, contrary to the monoclinic *P2*_1_ */c* space group of graftonite due to a difference in Fe oxidation and ionic radius.

**Figure 7:**
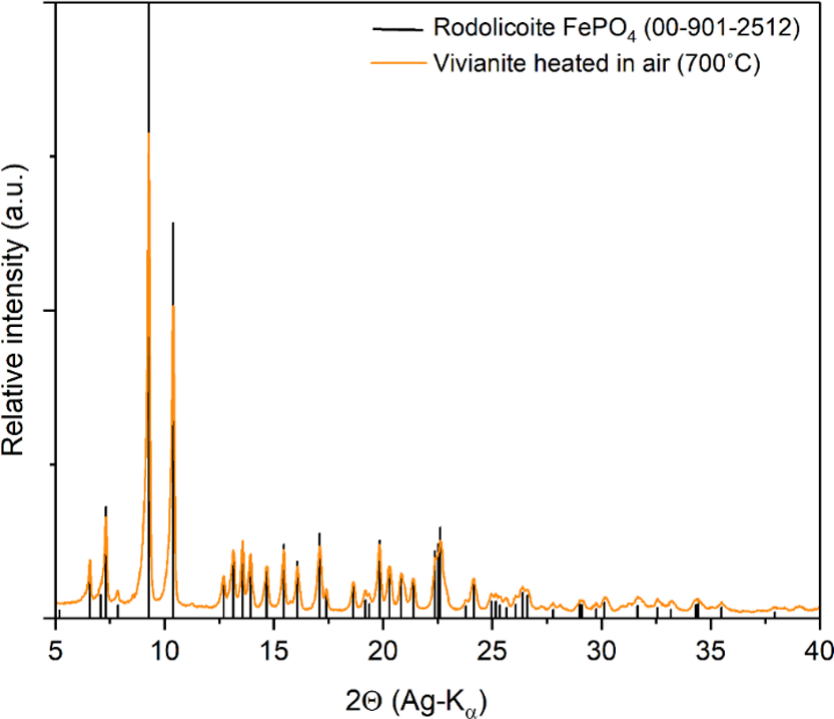
XRD pattern of the product of an experiment in which we heat-treated vivianite in air for 1 h at 700 °C (orange trace) matching the calculated XRD pattern of crystalline ferric phosphate (rodolicoite) from the COD database (00-901-2512)—black bars.

Our oxic heating experiments were not performed in situ, and thus, we only analyzed the final product. The results did not document the presence of meta vivianite at high temperatures, as it forms upon oxidation of vivianite at ambient to low temperatures.^5^ We do not know if an amorphous intermediate phase forms, but Huang et al.^10^ showed that when one starts with an amorphous ferric phosphate (Fe^3+^ PO_4_), rodolicoite forms at temperatures above 620 °C, similar to our results at 700 °C. In our case, the starting material was vivianite; thus, our transformation included the oxidation of Fe^2+^ to Fe^3+^ prior to the crystallization of rodolicoite.

To evaluate changes in oxidation states and geometry of iron during the anoxic/oxic experiments, we performed TEM-EELS measurements on graftonite, the amorphous intermediate Fe_3_(PO_4_)_2_, and rodicolite as shown in Figure S7, Supporting Information.

Figure S7 (Supporting Information) shows illustrative high-angle annular dark-field (HAADF) -STEM images of amorphous Fe_3_(PO_4_)_2_, graftonite, and rodolicoite (FePO_4_) and the corresponding O–K and Fe-L_2,3_ edge spectra. The O–K edge shows a prepeak feature (∼531 eV) for rodolicoite, whereas the other two (Fe^2+^ phases), graftonite and amorphous Fe_3_(PO_4_)_2_, lack this feature. This prepeak feature corresponds to electronic transitions from the O *1s* (core state) → O (*2p*) *–* Fe (*3d*) (hybridized states).^30^ The height of the prepeak is related to the occupancy of Fe 3d orbitals and Fe–O bond distances, Fe^3+^ phosphate (rodolicoite) distinctly shows this feature at ∼532 eV, whereas, according to literature, a Fe^2+^ phase such as LiFePO_4_ lacks it.^31^ The two major features of the Fe-L_2,3_ are the strong white lines L_3_ and L_2_ due to the spin-orbit splitting of the 2p core hole^31^ and separated by about 12 eV. Rodolicoite (Fe^3+^ in the tetrahedral site) is characterized by an L_3_ edge peak maximum at 711 eV, whereas amorphous Fe_3_(PO_4_)_2_ and graftonite are characterized by an L_3_ edge peak maximum at 709 eV. The amorphous Fe_3_(PO_4_)_2_ shows a shoulder feature at ∼711 eV. These Fe- L_2,3_ edges show a characteristic behavior with changing Fe oxidation states. Partial aerial oxidation of the vivianite sample can also lead to Fe^3+^ impurities in the annealed products. Therefore, to synthesize graftonite, special care must be taken to ensure the anoxicity of the system.

Our results show that the Fe^2+^ remains stable after heating, and the water autodecomposition does not occur if the sample is heated in an inert atmosphere, contrary to the results of Hanzel et al. that proposed oxidation to be taking place as a result of thermally driven autodecomposition of H_2_O molecules during heating of vivianite in the absence of air.^6^

## 4 Conclusions

Vivianite, when heated in anoxic conditions, notably undergoes structural transformation via two steps. First, vivianite dehydrates and decomposes above 250 °C into an anhydrous, still ferrous, but amorphous phase. Upon continued anoxic heating (>500 °C), this then recrystallizes to graftonite, a crystalline ferrous phosphate, Fe_3_(PO_4_)_2_, with a distinctly different structure from the initial vivianite. At this temperature, this second step is thermodynamically feasible, as evidenced by a crystallization molar enthalpy of −16.76 ± 0.26 kJ mol^–1^.

Our results document the major variations in structural hydration, crystal symmetry, Fe coordination, and morphology induced by this anoxic high-temperature-driven atomic rearrangement in the solids. Both dehydrated ferrous phases (graftonite and amorphous Fe_3_(PO_4_)_2_) exhibit a similar bulk shape, bubble’-like features in their electron micrographs and possess a porous structure, contrary to uniform and smooth-surfaced vivianite crystals. The transformation also reduces the molar volume of the initial vivianite by ∼ 3% but the overall shape of the initial vivianite crystals is preserved despite their shrinking volume. On the contrary, when the same initial vivianite is heated under oxic conditions, it completely oxidizes and crystallizes into a ferric phosphate phase, i.e., rodolicoite (FePO_4_), instead of the ferrous graftonite.

Although Fe-graftonite only forms under strictly anoxic conditions, it is stable in air once formed. Nevertheless, our data show that the transformation of vivianite can occur via different pathways depending on the oxygen fugacity and hydration of the system. Our insights also document a facile synthesis method to form thermodynamically stable high-temperature iron phosphate materials (graftonite, rodolicoite, amorphous Fe_3_(PO_4_)_2_) from synthetic vivianite, that is easily synthesized at ambient temperature and pressure. Graftonite and amorphous Fe_3_(PO_4_)_2_ are promising synthetic precursors to produce cathodic LiFePO_4_ battery materials. Their inherent porosity and high surface area may enhance Li^+^ diffusion dynamics and electrical conductivity, leading to an improved electrochemical performance. Nonetheless, it is crucial to carefully manage the synthesis process to prevent exposure to air and oxidation of Fe^2+^, ensuring stability and effectiveness of the final product. This understanding can pave the way for the development of advanced materials with tailored properties for specific applications.

In a broader context, our work also illuminates a direct link between crystal structures of vivianite and graftonite and rodolicoite and offers novel insights into the thermodynamic crystal phase stabilities of ferrous phosphates. This research not only contributes to the fundamental knowledge of mineralogy but also has practical implications for enhancing the performance and durability of materials in technological applications.

## Supplementary Material





## Notes

XRD calibration curves, preparation of samples for Fe–K edge XAS, powder XRD pattern of vivianite, Rietveld refinement details, temperature resolved in situ SEM micrographs, normalized Fe K-edge XANES spectra, Fe–K EXAFS data, shell-by-shell fits of k3-weighted EXAFS spectra, and HAADF—STEM and TEM-EELS images (PDF)
In situ SEM of vivianite heated up to 700 °C (AVI)

## ⊥

Present address: Chemical Sciences and Engineering Division, Argonne National Laboratory, 9700 S Cass Avenue, Lemont, Illinois 60439, United States

## #

Present address: University of Warsaw, Faculty of Biology, Ilji Miecznikowa 1, 02-096 Warsaw, Poland.
