# Supplementary material for: Temperature-Induced Phase Transitions of Vivianite: In Situ Analysis of a Redox-Driven Crystallization
Source: Inorg Chem. 2025 Aug 28;64(36):18227–36. doi: 10.1021/acs.inorgchem.5c02399 (PMC12442088; doi:10.1021/acs.inorgchem.5c02399)
Supplement: Supplementary file 1 [file ic5c02399_si_001.pdf]

Supporting Information

Temperature-Induced Phase Transitions of Vivianite: *In Situ* Analysis of a Redox-Driven Crystallization

Alice Paskin [⁋,†,,*], *Thaïs Couasnon* [⁋], *Roberts Blukis* [⁋,¥], *Jeffrey Paulo H. Perez* [⁋], *Stefan Reinsch*[⁑], *Vladimir Roddatis* [⁋], *Marcin Syczewski* [⁋,§], *Liane G. Benning* [⁋,‡,*]

AUTHOR ADDRESSES

[⁋] GFZ Helmholtz Centre for Geosciences, Telegrafenberg, 14473 Potsdam, Germany

[‡] Department of Earth Sciences, Freie Universität Berlin, Malteserstr. 74-100, 12249 Berlin, Germany

[¥] Leibniz-Institut für Kristallzüchtung (IKZ), Max-Born Str. 2, 12489 Berlin, Germany

[⁑] Federal Institute for Materials Research and Testing (BAM), Richard-Willstätter-Straße 11, 12489 Berlin, Germany

Corresponding author email(s)

[*] Liane G. Benning – benning@gfz.de; Alice Paskin – apaskin@anl.gov

Contents

Figure S1.	Calibration curve for a STOE high-temperature XRD furnace accessory
Section S1	Preparation of samples for Fe-K edge XAS spectroscopy
Figure S2	Indexed powder XRD pattern of synthetic vivianite
Table S2.	Atomic parameters of vivianite unit cell from Rietveld refinements
Table S3.	Atomic parameters of graftonite unit cell from Rietveld refinements
Figure S3	Temperature resolved *in situ* SEM micrographs of vivianite (20 – 700˚C)
Figure S4	SEM-EDX spectra of synthetic materials
Figure S5	Normalized Fe K-edge XANES spectra
Figure S6.	Fe K-edge XAS data
Table S3	Shell-by-shell fits of k^3-weighted EXAFS spectra
Figure S7	HAADF—STEM images and TEM-EELS data

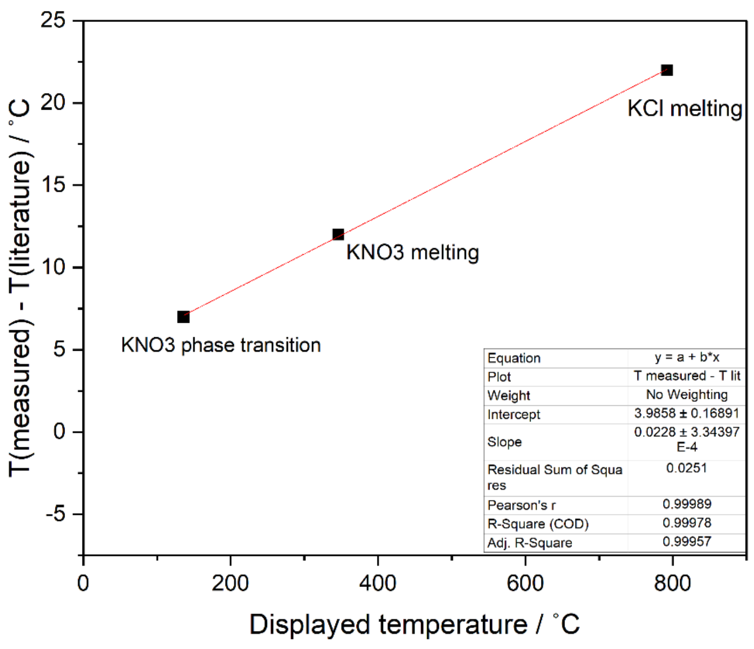

Figure S1. Calibration curve with phase transitions / melting points for a STOE high-temperature XRD furnace accessory, when using a heating rate of 10˚C/min.

Section S1. Preparation samples for Fe-K edge XAS spectroscopy

Pellet preparation and protocol for preventing and testing for sample oxidation

Pellets were prepared in a glovebox (97 % N_2, 3% H_2) at the GFZ Potsdam by mixing powdered samples with cellulose; the mixture was based on calculations using the XAFSmass software.[1] Pressed pellets were sealed inside 2 layers of single-sided 70-μm thick Kapton® polyimide tape, and then fixed onto a custom-made sample holder from the beamline. Inside the glovebox, the so prepared samples were sealed inside airtight Mylar® bags with an impulse heat sealer. These bags were transferred into and transported to the beamline inside an airtight, anaerobic jar (Oxoid™ AnaeroJar™, Thermo Fischer Scientific) filled with the glovebox atmosphere (97 % N_2, 3% H_2) and only removed and loaded at the beamline before XAS measurement.

Details of As K-edge XAS data collection

Iron K-edge XAS data were collected on the P65 undulator beamline of DESY German Electron Synchrotron (HASYLAB, DESY PETRA III, Hamburg, Germany). Incoming photon flux energy was modulated with a Si(111) double crystal monochromator, with an energy resolution of ~0.6 eV at the Fe K-edge and a beam size of 0.3 × 1.5 mm^2. The effective suppression of higher harmonics was achieved using Si-plane mirrors. The data were collected from -160 eV below the edge to +800 eV above. Spectra of the samples were acquired in transmission mode at room temperature, concomitantly with the spectrum of an Fe foil for energy calibration and alignment. Meanwhile, the spectra of the reference phases were collected at 20 K in an earlier beamtime session [2]. Multiple scans were collected for each sample depending on data quality (at least 3 scans). Changes in line shape and peak position indicative of beam-induced photoreduction were examined and no beam damage was observed during spectra collection. The first inflection point in the first derivative of the adsorption threshold of the Fe foil was calibrated at 7112 eV. X-ray

absorption near-edge structure (XANES) data handling and edge analyses were done on Athena software [3].

Shell-by-shell fitting procedure

Shell-by-shell fits of the EXAFS spectra of the samples (k-range = 2-11.5 Å^{-1}) and amorphous Fe phosphate references (k-range = 2-12.5 Å^{-1}) were performed from 1 to 3.5 Å in R+ΔR-space, while those for the crystalline Fe phosphate phases (k-range = 2-12.5 Å^{-1}) were conducted from 1 to 6.5 Å. All fits were performed using the SIXPack software[4] based on algorithms derived from IFEFFIT.[5] The fits included the interatomic distance (R), the coordination number (CN), the mean squared atomic displacement parameter (σ^2), and the change in threshold energy (ΔE_0) for one sample. Phase and amplitude functions for single and multiple scattering paths were calculated using FEFF6 [6] and included Fe-O, Fe-Fe, Fe-P and Fe paths derived from the structure of goethite[7], strengite[8] and vivianite[9]. In preliminary fits, the CN and σ^2 were found to be highly correlated, which produced high fit-derived standard errors in these fitting parameters. We constrained σ^2 to 0.01 and 0.009 Å^2 in the second shell fits of Fe-Fe and Fe-P paths for the samples to reduce the high correlations as done in a previous work [10]. For the crystalline vivianite samples, the σ^2(Fe-Fe) paths were constrained at 0.006 Å^2 [10]. Consistent with previous work, the passive electron reduction parameter (S_0^2) in each fit was set to 0.9 [11]. The goodness-of-fit was assessed based on the R-factor, which is defined as the mean square difference between the fit and the data on a point-by-point basis: R-factor = $\sum_i (data_i - fit_i)^2 / \sum_i (data_i)^2$. An R-factor < 0.05 is considered to reflect a reasonable fit [12]. The validity of the addition of all scattering paths was evaluated using the F-test for EXAFS [13].

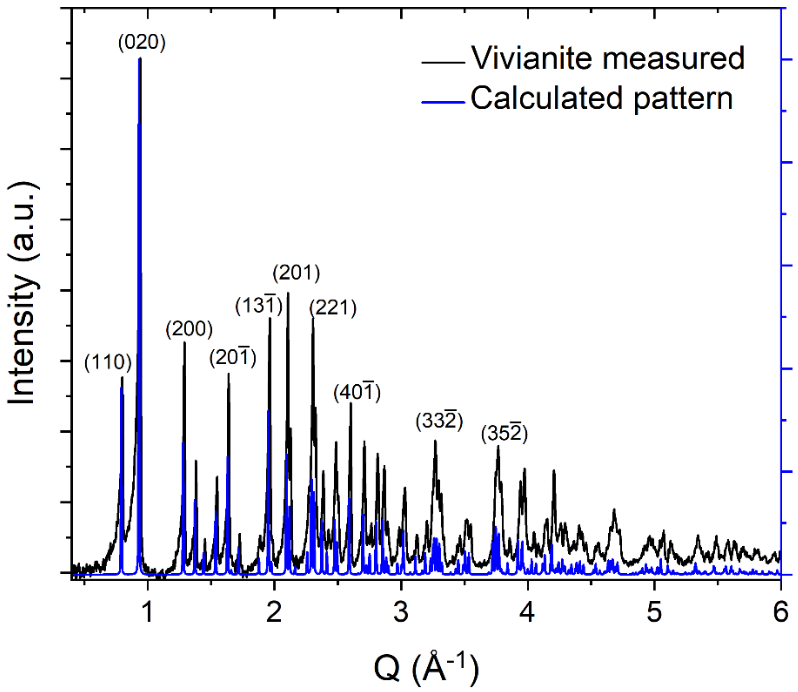

Figure S2. Indexed powder XRD pattern of synthetic vivianite from a *cif* file. [9]

Table S1. Atomic parameters of vivianite unit cell from Rietveld refinements

Atom	x	y	z	U_{iso}
Fe1	0.00000	0.00000	0.00000	0.0187(12)
Fe2	0.00000	0.38971(17)	0.00000	0.0137(8)
P1	0.3152(4)	0.00000	0.3744(10)	0.0125(12)
O1	0.1547(8)	0.00000	0.3678(19)	0.0298(29)
O2	0.3902(10)	0.00000	0.7038(20)	0.029(3)
O3	0.3409(5)	0.0966(5)	0.2176(12)	0.0325(21)
O4	0.1040(5)	0.1184(4)	0.7986(11)	0.0182(20)
O5	0.3986(6)	0.2245(4)	0.7107(13)	0.0189(20)

Table S2. Atomic parameters of graftonite unit cell from Rietveld refinements

Atom	x	y	z	U_{iso}
Fe1	0.9271(9)	0.1147(8)	0.8669(12)	0.0174(25)
Fe2	0.7233(9)	0.0793(7)	0.3361(16)	0.0230(28)
Fe3	0.3615(9)	0.1943(7)	0.1167(14)	0.0164(23)
P4	0.0962(15)	0.1382(10)	0.3943(25)	0.009(4)
P5	0.6034(15)	0.0887(10)	0.7962(22)	0.001(3)
O6	0.0699(31)	0.0581(25)	0.175(5)	0.008(9)
O7	0.48665	0.18172	0.82108	0.0206
O8	0.95193	0.20663	0.40837	0.0071
O9	0.70576	0.13144	0.62498	0.0161
O10	0.226(4)	0.2267(30)	0.376(6)	0.037(12)
O11	0.7275(31)	0.0845(27)	0.009(5)	0.008(9)
O12	0.1220(27)	0.0598(26)	0.594(5)	0.000(8)
O13	0.53020	0.03050	0.76400	0.0550

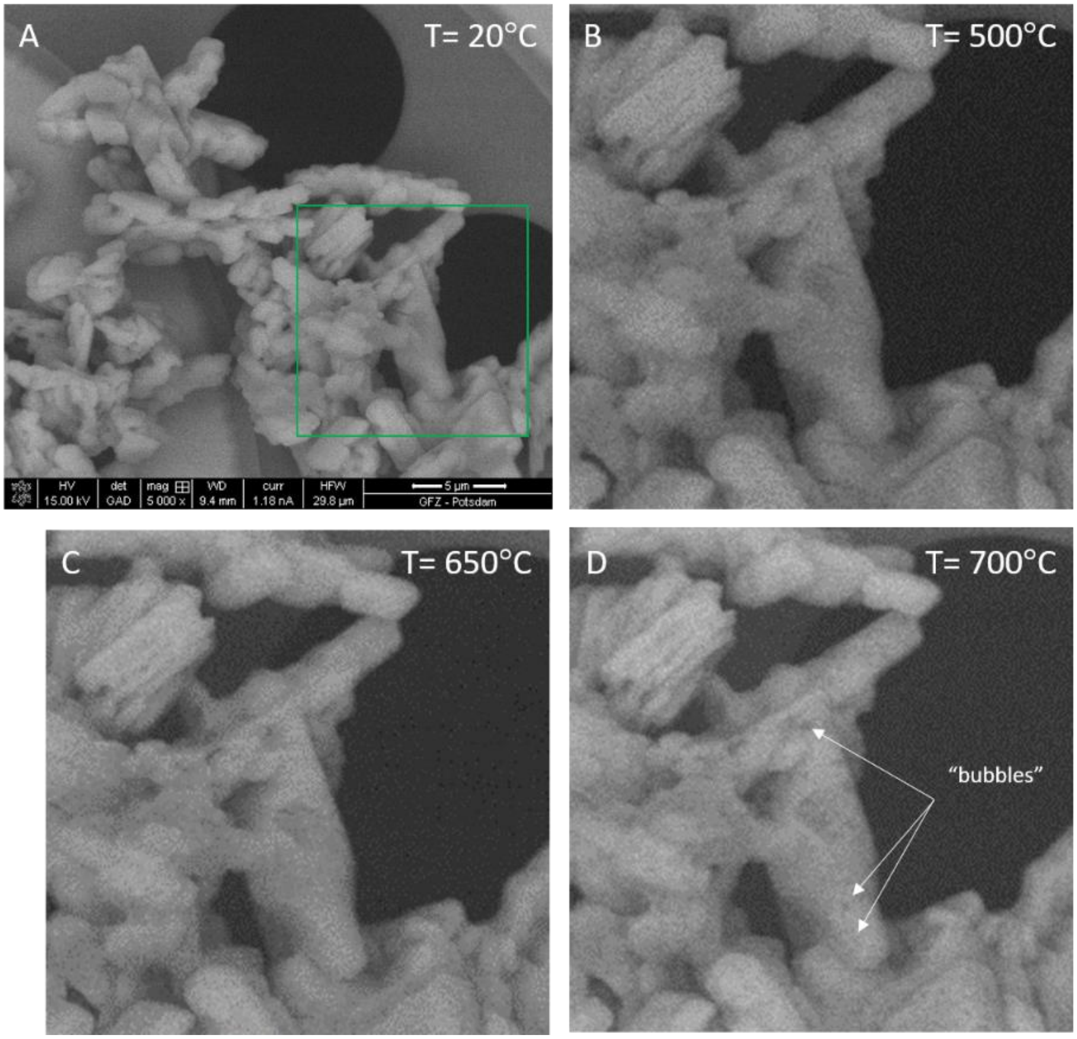

Figure S3. Selected snapshots of selected time frames from video recorded during the *in situ* SEM experiments (A) SEM image recorded prior to initiating the heating. (B-D) zoomed images at different temperatures from the area marked in (A). White arrows point out the development of the porous, "bubble"- like features.

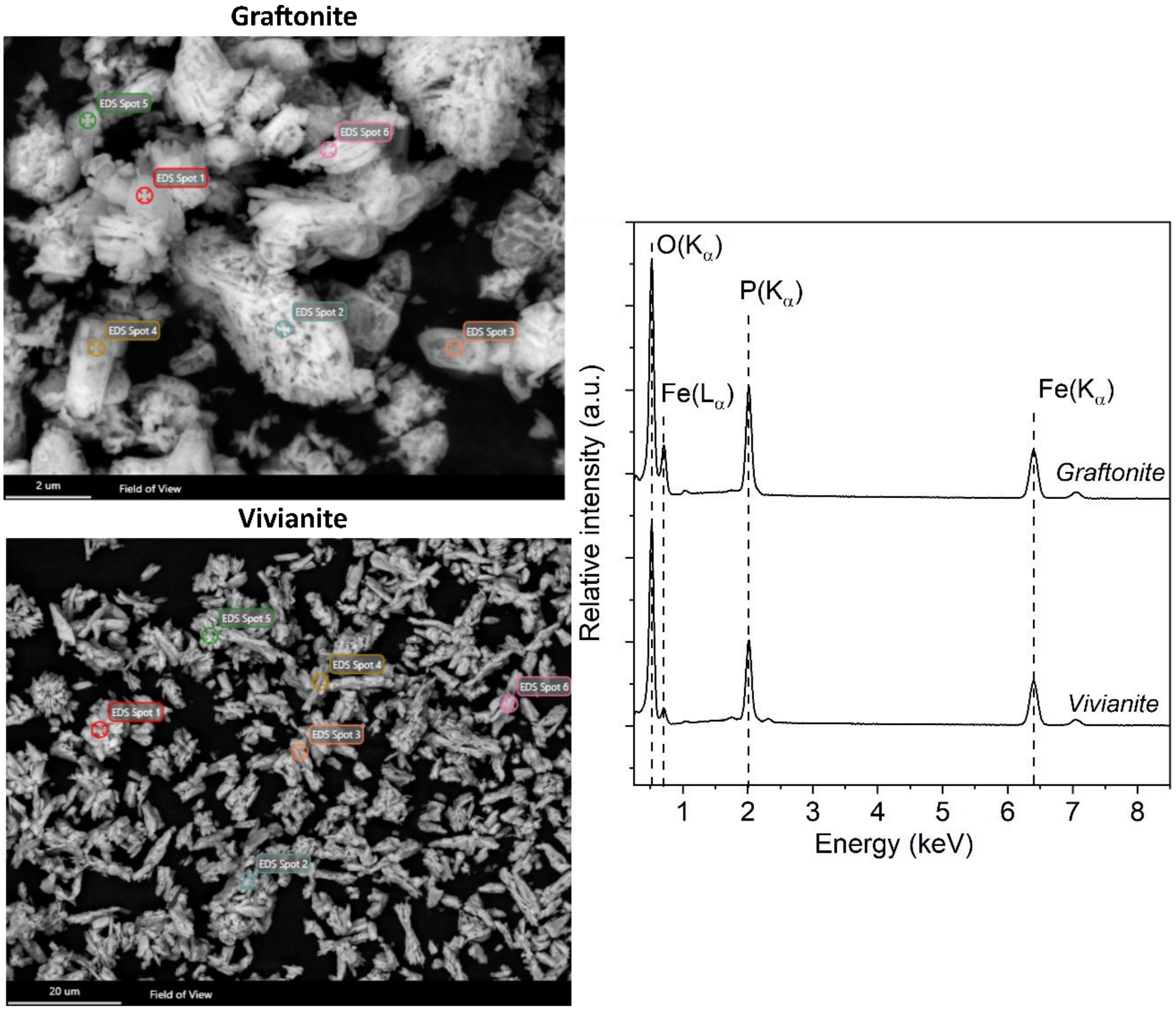

Figure S4. SEM-images and EDX spectra of graftonite (top) and vivianite (bottom) with each EDX spectrum corresponding to an average of EDX analysis from 5 different spots, as shown in each image

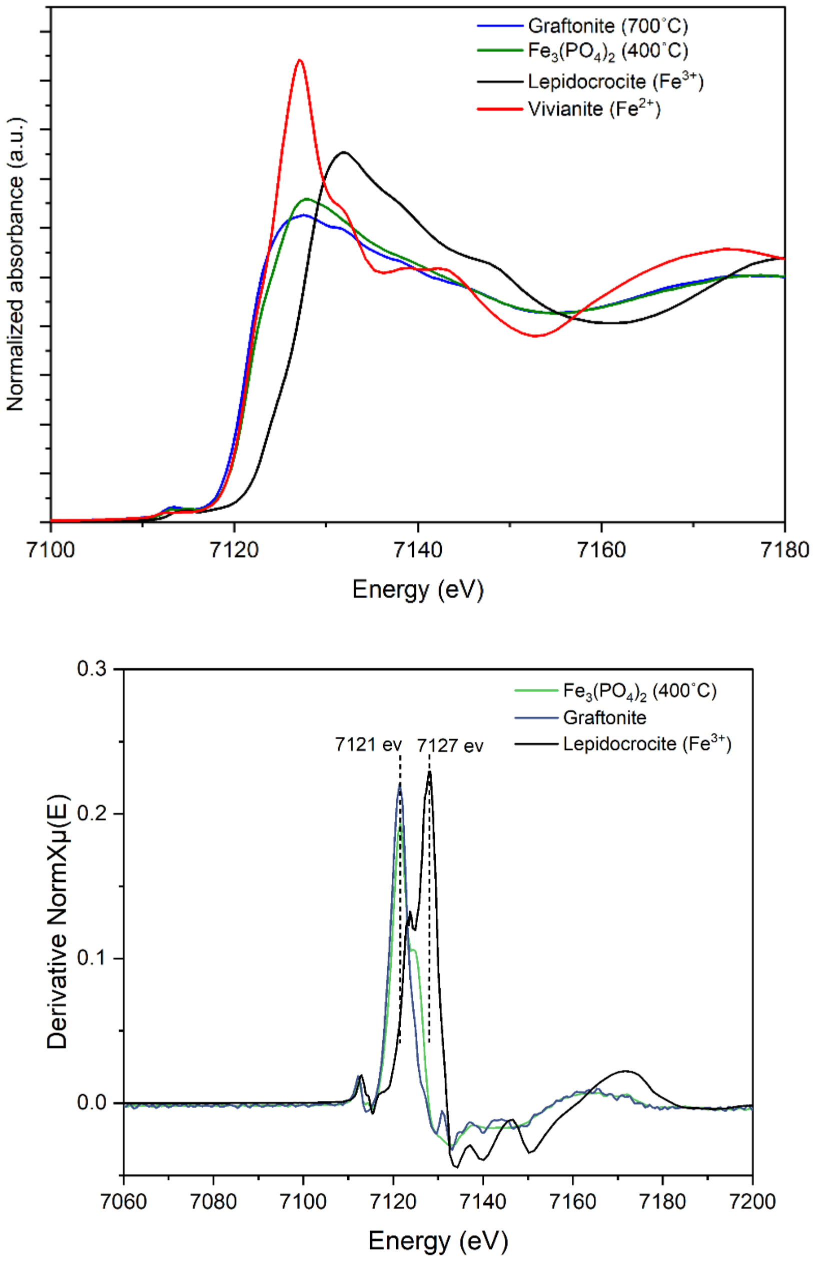

Figure S5. (*Top*) Normalized Fe K-edge XANES spectra collected for lepidocrocite (γ-Fe(O)OH (Fe^{3+} reference), the at 400˚C collected amorphous ferrous intermediate ($Fe_3(PO_4)_2$), vivianite and graftonite (700˚C); (*Bottom*) First derivative of χ(k) for amorphous $Fe_3(PO_4)_2$ (400˚C), graftonite (mainly Fe^{2+}) and lepidocrocite (Fe^{3+} reference)

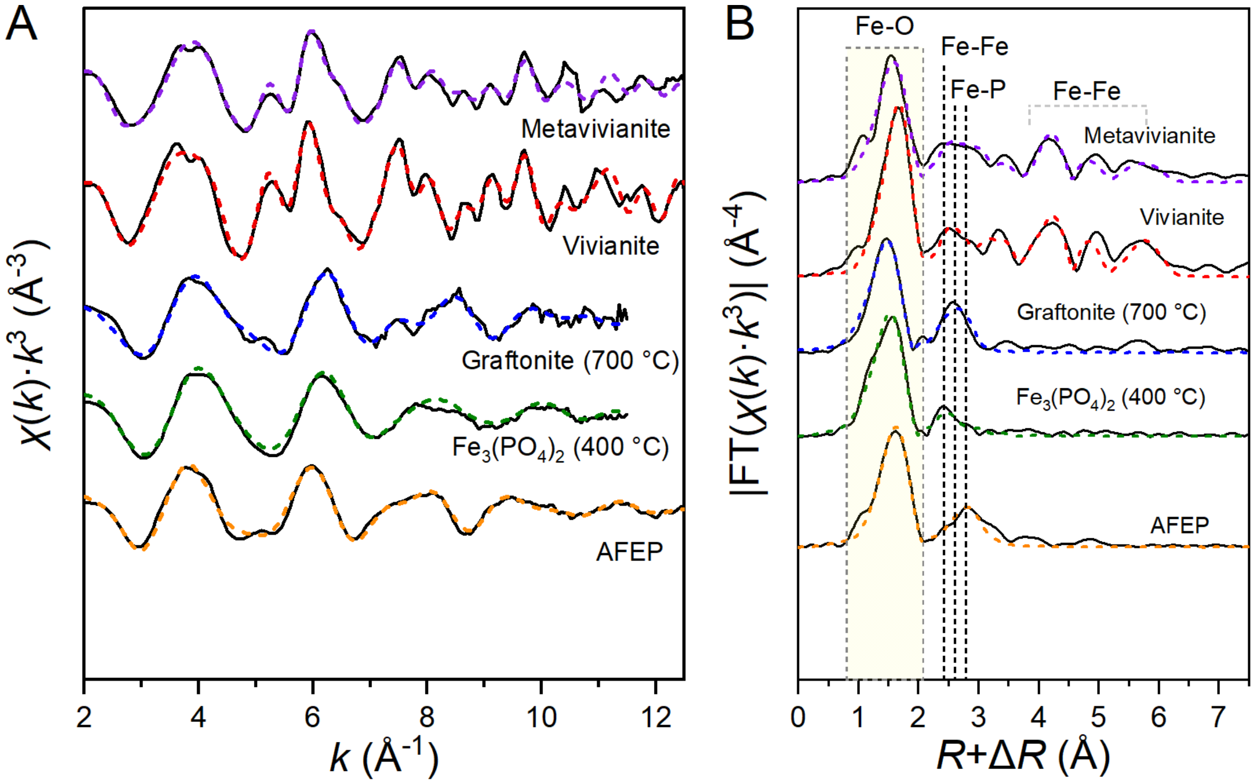

Figure S6. Fe K-edge XAS data for the end product graftonite (700 °C) – *blue trace* and the amorphous $Fe_3(PO_4)_2$ intermediate phase (400 °C) – *green trace* at RT as well as the reference phases (metavivianite – *purple trace*, vivianite – *red trace*, AFEP ($Fe_3(PO_4)_2{\cdot}4.7H_2O$) [2] – *green trace*) at 20 K: (A) k^3-weighted EXAFS spectra and (B) Fourier-transformed EXAFS spectra. The shell-by-shell fitting output (red dashed lines) are superimposed on the experimental data (black solid lines). See Table S3 for fitting results.

Table S3. Shell-by-shell fits of k^3-weighted EXAFS spectra of graftonite and the $Fe_3(PO_4)_2$ intermediate.

Sample	Atomic pair	*CN*	*R* (Å)	σ^2 (Å^2)	ΔE_0 (eV)	n_{idp}/n_{var}	χ_v^2	R_f
Graftonite (700 °C)	Fe-O	3.7 (0.5)	1.99 (0.01)	0.012 (0.002)	-3.9 (1.4)	15/8	39	0.019
	Fe-Fe1	0.7 (0.4)	3.02 (0.03)	*0.010*				
	Fe-P	1.1 (0.6)	3.21 (0.04)	*0.008*				
$Fe_3(PO_4)_2$ (400 °C)	Fe-O	4.2 (0.6)	2.03 (0.01)	0.012 (0.002)	0.4 (1.4)	15/8	175	0.023
	Fe-Fe	0.7 (0.3)	3.01 (0.03)	*0.010*				
	Fe-P	0.8 (0.4)	3.35 (0.05)	*0.008*				
Reference phases								
$Fe_3(PO_4)_2 \cdot 4.7H_2O$ (AFEP)	Fe-O	5.5 (0.6)	2.10 (0.01)	0.010 (0.001)	3.6 (1.0)	17/7	381	0.018
	Fe-P	3.7 (1.1)	3.34 (0.01)	0.011 (0.003)				
Vivianite, $Fe_3(PO_4)_2 \cdot 8H_2O$	Fe-O2	6.4 (0.6)	2.13 (0.01)	0.008 (0.001)	2.0 (0.9)	36/18	150	0.027
	Fe-Fe1	1.1 (0.3)	3.03 (0.02)	*0.006*				
	Fe-P	1.7 (0.6)	3.34 (0.03)	*0.008*				
	Fe-O	5.9 (1.7)	3.98 (0.02)	σ^2 (Fe-O1)				
	Fe-O-O	6.3 (2.6)	4.45 (0.04)	σ^2 (Fe-O1)				
	Fe-Fe2	4.8 (0.8)	4.69 (0.01)	σ^2 (Fe-Fe1)				
	Fe-Fe3	4.2 (0.9)	5.22 (0.01)	σ^2 (Fe-Fe1)				
	Fe-Fe4	7.5 (1.5)	6.25 (0.01)	σ^2 (Fe-Fe1)				
Metavivianite, $Fe^{II}_{3-x}Fe^{III}_{x}(PO_4)_2(OH)_x \cdot (8-x)H_2O$	Fe-O	6.7 (0.9)	2.09 (0.01)	0.013 (0.002)	1.0 (1.1)	36/16	123	0.049
	Fe-Fe1	0.6 (0.3)	3.00 (0.02)	*0.006*				
	Fe-P	2.3 (0.6)	3.29 (0.02)	*0.008*				
	Fe-O2	7.1 (2.4)	4.00 (0.03)	σ^2 (Fe-O1)				
	Fe-Fe2	3.4 (0.6)	4.66 (0.01)	σ^2 (Fe-Fe1)				
	Fe-Fe3	5.8 (1.7)	5.22 (0.02)	$2\sigma^2$ (Fe-Fe1)				
	Fe-Fe4	9.1 (3.0)	6.26 (0.03)	$2\sigma^2$ (Fe-Fe1)				

Note: CN – coordination number, R – interatomic distance, σ^2 – mean-squared displacement, ΔE_0 – change in threshold energy, n_{idp} – no. of independent data points, n_{var} – no. of variables, χ_v^2 – reduced CHI square, and R_f – R-factor. Values given without fit uncertainty (in parenthesis) were fixed. The amplitude reduction factor (S_0^2) was set to 0.9 in all fits

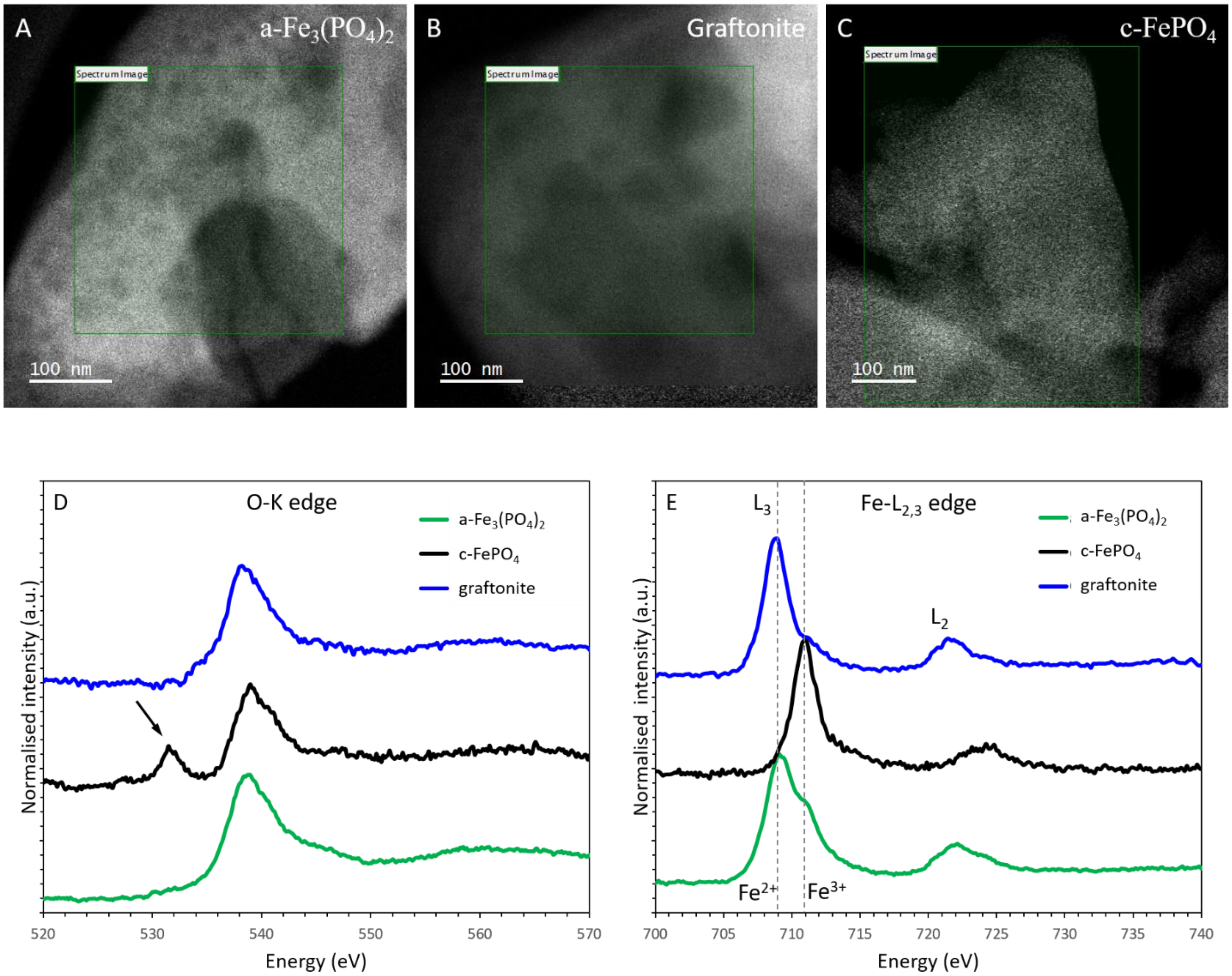

Figure S7. O-K edge and Fe-$L_{2,3}$ spectra of the amorphous $Fe_3(PO_4)_2$ – green trace, graftonite – blue trace and rodolicoite ($FePO_4$) – black trace. The corresponding spectrum images were collected from areas marked with green contours. The black arrow in (D) marks the visible pre-edge feature in O-K edge EELS spectrum of rodolicoite – black trace (a characteristic of Fe^{3+} compounds)

References

1. Klementiev, K., and Chernikov, R., *J. Phys. Conf. Ser.* (2016) **712**, 012008
2. Paskin, A.*, et al.*, *Journal of the American Chemical Society* (2023) **145** (28), 15137
3. Ravel, B., and Newville, M., *Journal of Synchrotron Radiation* (2005) **12**, 537
4. Webb, S. M., *Phys. Scr.* (2005) **T115** (T115), 1011
5. Newville, M., *J. Synchr. Radiat.* (2001) **8** (Pt 2), 322
6. Rehr, J. J.*, et al.*, *Phys. Rev. Lett.* (1992) **69** (23), 3397
7. Rakovan, J.*, et al.*, (1999) **84** (5-6), 884
8. Taxer, K., and Bartl, H., *Crystal Research and Technology* (2004) **39** (12), 1080
9. Capitelli, F.*, et al.*, *Zeitschrift Fur Kristallographie-Crystalline Materials* (2012) **227** (2), 92
10. Bae, S.*, et al.*, *Environmental Science & Technology* (2018) **52** (18), 10647
11. Mikutta, C.*, et al.*, *Geochimica et Cosmochimica Acta* (2014) **140**, 708
12. Kelly, S. D.*, et al.*, Analysis of Soils and Minerals Using X-ray Absorption Spectroscopy. In *Methods of Soil Analysis Part 5—Mineralogical Methods*, (2008), pp 387
13. Downward, L.*, et al.*, *AIP Conference Proceedings* (2007) **882** (1), 129
